# Patterns of comorbidities, clinical course, and impact of the ABC Pathway for Integrated Care in patients with atrial fibrillation: a report from the prospective Optimal Thromboprophylaxis in Elderly Chinese Patients with Atrial Fibrillation (ChiOTEAF) Registry

**DOI:** 10.1093/ehjqcco/qcaf014

**Published:** 2025-04-15

**Authors:** Marta Mantovani, Tommaso Bucci, Jacopo F Imberti, Steven H M Lam, Agnieszka Babinska, Giuseppe Boriani, Yutao Guo, Gregory Y H Lip, the ChiOTEAF Registry Investigators, Gregory Y H Lip, Xiaoying Li, Yutang Wang, Changsheng Ma, Shu Zhang, Congxin Huang, Jiefu Yang, Meilin Liu, Yutao Guo, Guangliang Shan, Taixiang Wu, Chen Yao, Changsheng Ma, Congchun Huang, Cuntai Zhang, Dang Aiming, Dawei Qian, Fakuan Tang, Fang Wu, Feng Liu, Gexin Zhu, Guo Yutao, Guorong Xi, Heng Dou, Hou Cuihong, Hua Li, Hui Han, Huiliang Liu, Jian Kong, Junxia Li, Liang Zaoguang, Liangyi Si, Liu Meilin, Liu Yanxia, Liu Yu, Liu Zhiming, Luo Ma, Ming Li, Qian Xiao, Qingwei Chen, Qiong Chen, Ren Xuejun, Shan Zhaoliang, Shi Xiangming, Shilian Hu, Song Bai, Tianchang Li, Wang Lijuan, Wu Qiang, Xianghu Wang, Xiaojuan Bai, Xiaoming Wang, Xinchun Yang, Xuan He, Xuejun Liu, Yan Li, Yang Jiefu, Yong Wang, Yunmei Yang, Zeng Yuan, Zhang Shu, Zhang Wei, Zhanyi Lin

**Affiliations:** Liverpool Centre for Cardiovascular Science at University of Liverpool, Liverpool John Moores University and Liverpool Heart and Chest Hospital, Liverpool, L7 8TX, UK; Cardiology Division, Department of Biomedical, Metabolic and Neural Sciences, University of Modena and Reggio Emilia, Policlinico di Modena, Modena 41125, Italy; Clinical and Experimental Medicine PhD Program, University of Modena and Reggio Emilia, Modena 41125, Italy; Liverpool Centre for Cardiovascular Science at University of Liverpool, Liverpool John Moores University and Liverpool Heart and Chest Hospital, Liverpool, L7 8TX, UK; Department of Clinical Internal, Anaesthesiologic and Cardiovascular Sciences, Sapienza University of Rome, Rome 00161, Italy; Liverpool Centre for Cardiovascular Science at University of Liverpool, Liverpool John Moores University and Liverpool Heart and Chest Hospital, Liverpool, L7 8TX, UK; Cardiology Division, Department of Biomedical, Metabolic and Neural Sciences, University of Modena and Reggio Emilia, Policlinico di Modena, Modena 41125, Italy; Clinical and Experimental Medicine PhD Program, University of Modena and Reggio Emilia, Modena 41125, Italy; Liverpool Centre for Cardiovascular Science at University of Liverpool, Liverpool John Moores University and Liverpool Heart and Chest Hospital, Liverpool, L7 8TX, UK; Department of Cardiology and Angiology, Silesian Center for Heart Diseases, 41-800 Zabrze, Poland; Department of Cardiology and Electrotherapy, Faculty of Medical Sciences in Zabrze, Medical University of Silesia, 40-055 Katowice, Poland; Cardiology Division, Department of Biomedical, Metabolic and Neural Sciences, University of Modena and Reggio Emilia, Policlinico di Modena, Modena 41125, Italy; Department of Pulmonary Vessel and Thrombotic Disease, Sixth Medical Centre, Chinese PLA General Hospital, Beijing 100853, China; Liverpool Centre for Cardiovascular Science at University of Liverpool, Liverpool John Moores University and Liverpool Heart and Chest Hospital, Liverpool, L7 8TX, UK; Department of Clinical Medicine, Aalborg University, Aalborg 9000, Denmark

**Keywords:** Atrial fibrillation, Asian registry, Clinical phenotypes, Comorbidities, Integrated approach, Outcome

## Abstract

**Aims:**

To identify comorbidity patterns in elderly Chinese patients with atrial fibrillation (AF), their clinical course, and the effectiveness of the Atrial fibrillation Better Care (ABC) pathway adherence among these phenotypes.

**Methods and results:**

From the ChiOTEAF Registry, we performed a latent class analysis based on 16 cardiovascular (CV) and non-CV conditions. The association between classes of patients, management, and outcomes was evaluated. The primary outcome was a composite of all‐cause death and major adverse CV events. We assessed the impact of ABC adherence on outcomes in the whole cohort and among phenotypes. We included 4765 AF patients [median age 77 (68–83) years, 39.1% females]. Four phenotypes were identified: (i) low complexity (48.9%); (ii) atherosclerotic (19.3%); (iii) heart failure (19.4%); and (iv) high complexity (12.3%). During a 1-year follow-up, compared to the ‘low complexity’ class, the risk of adverse events was higher in ‘high complexity’ [aOR, 95% confidence interval (CI): 3.20, 2.21–4.66] and ‘heart failure’ classes (aOR, 95% CI: 1.50, 1.04–2.17). Among 2654 patients [median age 75 (66–81) years, 43.3% females] with available information to assess the ABC pathway, 1094 (41.2%) were adherent. ABC pathway adherence was associated with a lower risk of adverse events (aOR, 95% CI: 0.37, 0.20–0.65). On interaction analysis, its beneficial effect was similar across different clinical phenotypes (Pint = 0.122).

**Conclusion:**

Different clinical phenotypes can be identified in Asian AF patients, with specific patterns of comorbidities and different risks of adverse events. Full ABC pathway adherence was associated with improved outcomes, regardless of the clinical phenotype.

## Graphical Abstract

**Graphical Abstract:**
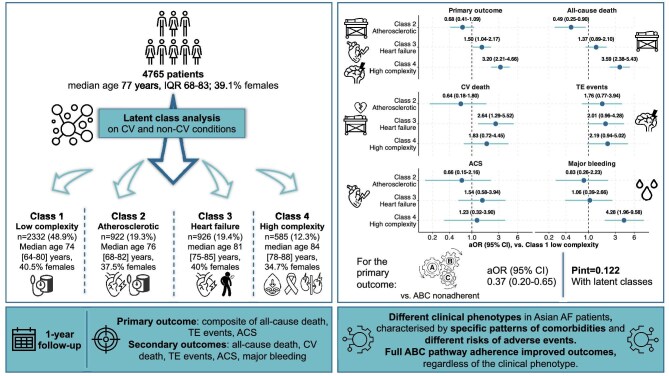

Key Learning Points **What is already known**

- Due to the ageing of the general population, atrial fibrillation (AF) is commonly associated with other concurrent chronic conditions, including cardiovascular (CV) and non-CV diseases leading to clinically complex phenotypes.
- The distinctive phenotypes significantly influence both management strategies and patient prognosis.

**What this study adds**

- In elderly Asian patients with AF, heterogeneous clinical phenotypes, each characterised by unique patterns of comorbidities, can be clearly distinguished.
- The beneficial impact of the Atrial fibrillation Better Care pathway adherence is similar across the various clinical phenotypes, reinforcing the importance of tailored approaches to target the different patterns of comorbidities.

## Introduction

Due to the ageing of the general population, the prevalence and incidence of atrial fibrillation (AF) are increasing, in association with other concurrent chronic conditions, including cardiovascular (CV) and non-CV diseases leading to clinically complex phenotypes of such patients.^1^ Multimorbidity, defined as the presence of at least 2 long-term conditions, poses a significant challenge and is a growing concern in the management of AF patients.^2,3^ Indeed, multimorbidity has a detrimental effect on AF patients, being associated with low use of oral anticoagulants (OAC), high OAC discontinuation and with an increased risk of adverse events.^4–6^

Large-scale registries and randomized trials have shown that holistic or integrated care management based on the Atrial fibrillation Better care (ABC) pathway, i.e. avoid stroke with anticoagulation; symptom reduction with rate or rhythm control; and CV risk factor and comorbidity management, including lifestyle changes, was effective in reducing the risk of adverse events, in patients with AF even when they were affected by multimorbidity.^7–15^ Indeed, current AF guidelines stress the role of an integrated approach to actively address comorbidities and risk factors.^16–20^

Of note, besides the number of concurrent conditions, there is increasing awareness that comorbidities tend to aggregate in different clusters, with various combinations of diseases.^2^ Latent class analysis (LCA) is a statistical technique that seeks to identify subgroups within a population characterized by homogeneous combinations of a set of prespecified variables. The effectiveness of this method in distinguishing clinical phenotypes and patterns of comorbidities has been previously proved in various cohorts.^21,22^ However, to date, most studies have been conducted in Western populations,^23,24^ leaving a critical gap in understanding how comorbidity patterns may shape care and outcomes in Asian AF patients, who are characterized by various unique features, including different prevalence of risk factors and concomitant diseases.^25^

Thus, this study aimed to identify various phenotypes from a large real-world cohort of elderly Chinese patients based on the presence of comorbidities, and to assess the association with choices of care and the risk of adverse events. Second, we aimed to analyse the ABC pathway adherence and its impact on the risk of adverse events in the various clinical phenotypes.

## Methods

### Study design

The design and the primary results of the Optimal Thromboprophylaxis in Elderly Chinese Patients with Atrial Fibrillation (ChiOTEAF) registry have been previously published.^26,27^ Briefly, this is a prospective, observational, multicentre nationwide registry held in 44 sites from 20 provinces in China. During the study period, between October 2014 and December 2018, patients with a documented AF episode within the last 12 months who provided written informed consent were consecutively enrolled. At baseline, investigators collected data regarding demographics, comorbidities, and medications for all patients enrolled, and reported this information in an electronic form. The study protocol was approved by the Central Medical Ethical Committee of Chinese PLA General Hospital, Beijing, China (approval no S2014-065- 01) and by the local institutional review boards at each participating centre. The study was performed according to the principles of the Declaration of Helsinki. For the purpose of this analysis, patients with available information concerning the conditions used to perform the clustering analysis were included, as detailed below.

### Latent class analysis

The term ‘latent’ refers to the fact that this method identifies classes that cannot be directly observed by the investigators. LCA calculates the probability of each participant belonging to each latent class.^28^ To define these latent classes, we included the following clinical conditions: hypertension, diabetes, lipid disorders, coronary artery disease (CAD), heart failure (HF), stroke, intracranial haemorrhage, extracranial bleeding, peripheral artery disease (PAD), chronic kidney disease (CKD), chronic obstructive pulmonary disease (COPD), dementia, anaemia, malignancy, thyroid disease (including both hyperthyroidism and hypothyroidism), and liver disease, as recorded by investigators at baseline in the electronic case form. All the cases with missing values were excluded.

The best number of classes was chosen based on the lowest values of the Bayesian Information Criterion and the consistent Akaike Information Criterion, and on clinical judgment. Then, each patient was assigned to one latent class, according to the highest modal posterior probability of membership. The groups were named according to the most relevant clinical characteristics, and the most prevalent comorbidities.

### Clinical scores and ABC pathway

The CHA_2_DS_2_-VASc, CHA_2_DS_2_-VA, and HAS-BLED scores were calculated as per definitions.^29–31^ Adherence to the ABC pathway was retrospectively assessed using baseline data, as previously reported.^13^ Briefly, patients were considered adherent to the single criterion if:

- ‘A’ criterion: if patients were properly prescribed with OAC according to their thromboembolic risk. In details, male patients with a CHA_2_DS_2_-VASc score ≥1 and female patients with a CHA_2_DS_2_-VASc score ≥2 receiving OACs, and male patients with CHA_2_DS_2_-VASc score = 0 and female patients with CHA2DS_2_-VASc score = 1 not receiving OACs were considered ‘A’ adherent.
- ‘B’ criterion: if patients had an EHRA score of I (no symptoms) or II (mild symptoms) at baseline.^32^
- ‘C’ criterion: if patients received the optimal treatment for the most common comorbidities (i.e. hypertension, CAD, PAD, HF, stroke, diabetes, and lipid disorders). The disease-specific treatments considered for the various conditions were the following: (i) for hypertension: angiotensin-converting enzyme inhibitors (ACEi) or angiotensin receptor blockers (ARB), calcium-channel inhibitors, diuretics, or beta-blockers; (ii) for CAD: ACEi or ARBs, beta-blockers, and statins; (iii) for PAD: statins; (iv) for previous stroke: statins; (v) for HF: ACEi or ARBs and beta-blockers; (vi) for diabetes: insulin or oral glucose lowering drugs; and (vii) for lipid disorders: statins.

Patients who fulfilled all the criteria were defined as ABC pathway adherent, otherwise they were defined as ABC pathway nonadherent. We also divided the cohort based on the number of fulfilled criteria into (i) 0–1 ABC criteria adherent; (ii) 2 ABC criteria adherent; and (iii) 3 ABC criteria adherent.

### Follow-up and adverse events

Clinical follow-up data were collected and reported by local investigators through patient visits, chart reviews, and/or telephonic contacts. Follow-up visits were regularly performed 12 months after enrolment.

The primary outcome was a composite of all-cause death, acute coronary syndrome (ACS), and any thromboembolic (TE) events. Secondary exploratory outcomes were the single components of the primary outcomes, CV death, and major bleeding. Major bleeding was defined as clinically overt bleeding accompanied by at least one of a decrease in the blood haemoglobin level ≥2.0 g/dL over 24 h, the need for a transfusion ≥2 units of packed red cells, or for corrective surgery, or bleeding at a critical site (i.e. extracranial, intraspinal, intraocular, pericardial, intraarticular, intramuscular with compartment syndrome, or retroperitoneal).^33^

### Statistical analysis

Continuous variables are described as median and interquartile range (IQR), and compared using appropriate non-parametric tests, while categorical variables are reported as counts and percentages and compared using the Chi-square test.

Univariable and multivariable logistic regression analyses were used to investigate the following among different latent classes:

- The odds of receiving various antithrombotic treatments, including
  - OAC;
  - Non-vitamin K antagonist oral anticoagulant (NOAC) vs. vitamin K antagonist (VKA);
  - Any antiplatelet (APT) therapy;
  - Dual antithrombotic therapy (DAT);
- The likelihood of undergoing:
  - Rate control strategies, defined as the prescription of any among beta-blockers, calcium-channel blockers, or digoxin;
  - Rhythm control strategies, defined as the use of antiarrhythmic drugs, any pharmacological or electrical cardioversion, or catheter ablation.
- The risk of primary and secondary outcomes.

Moreover, only in patients with available information needed for the ABC pathway adherence assessment, we used logistic regression to examine:

- The association between the ABC pathway adherence (ABC adherent vs. ABC nonadherent) and the risk of the primary and secondary outcomes;
- The effect of the increased number of ABC criteria fulfilled on the risk of adverse events (i.e. 0–1 ABC criteria vs. 2 or 3 ABC criteria); and
- The effect of ABC pathway adherence on the risk of adverse events, across various clinical phenotypes.

All results from the logistic regression analyses were reported as odds ratio (OR), with 95% confidence interval (CI).

The model used for the multivariable regression analyses on different antithrombotic treatments and adherence to the ABC pathway was adjusted for CHA_2_DS_2_-VASc score and the type of AF.

The model used for the multivariable regression analyses on the risk of adverse events among latent classes was adjusted for age, sex, the use of OAC, and the type of AF. CV or non-CV diseases were not included in this model, as they were used in the LCA to define the classes. All analyses were performed using R (version 4.3.2), LCA was performed using the ‘poLCA’ package. A two-sided *P* < 0.05 was considered statistically significant in all the analyses.

## Results

**Figure 1:**
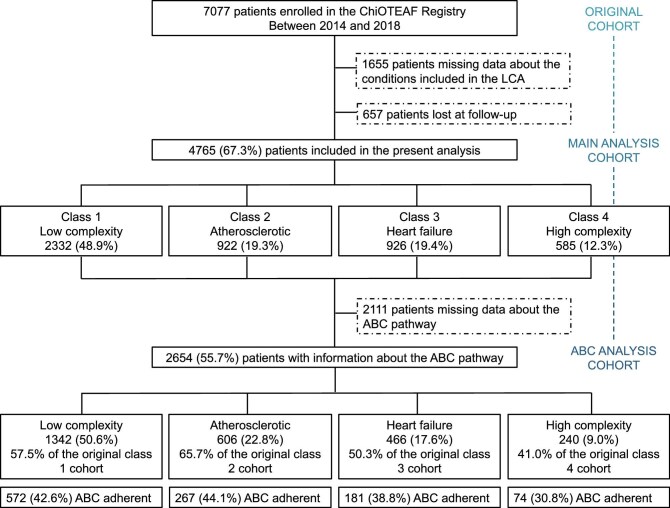
Flow-chart of the study. **Legend:** ABC, atrial fibrillation better care; ChiOTEAF, Optimal Thromboprophylaxis in Elderly Chinese Patients with Atrial Fibrillation; and LCA, latent class analysis.

From the original 7077 patients, 4765 (67.3%) patients (median age 77 years, IQR 68–83; 39.1% females) with complete data about the comorbidities used for the LCA and follow-up information were included in the present analysis. The flow-chart of the present analysis is reported in *Figure 1*.

### Classes of patients

**Figure 2:**
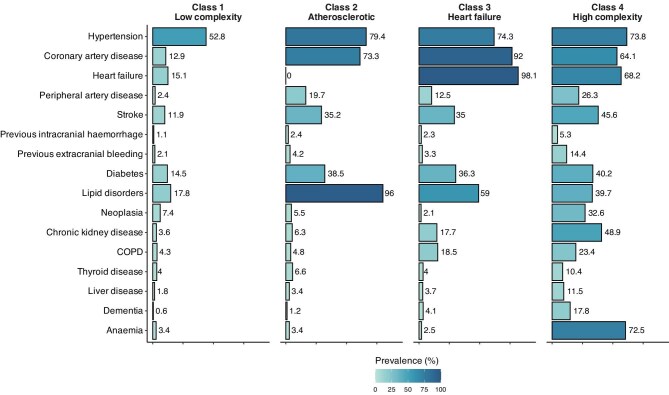
Prevalence of comorbidities according to latent classes. **Legend**: COPD, chronic obstructive pulmonary disease.

Baseline clinical characteristics of patients according to latent classes are summarised in *Table 1*. Moreover, a graphical representation of the prevalence of the 16 conditions included in the LCA is shown in *Figure 2*.

**Table 1:** Baseline characteristics according to latent classes

	Class 1	Class 2	Class 3	Class 4	
	Low complexity	Atherosclerotic	Heart failure	High complexity	*P* value
*N* (%)	2332 (48.9)	922 (19.3)	926 (19.4)	585 (12.3)	
Females, *n* (%)	945 (40.5)	346 (37.5)	370 (40.0)	203 (34.7)	0.046
Age (years), median [IQR]	74 [64–80]	76 [68–82]	81 [75–85]	84 [78–88]	<0.001
Comorbidities (*n*), median [IQR]	2 [1–2]	4 [3–4]	5 [4–6]	6 [5–7]	<0.001
Hypertension, *n* (%)	1231 (52.8)	732 (79.4)	688 (74.3)	432 (73.8)	<0.001
Lipid disorders, *n* (%)	415 (17.8)	885 (96.0)	546 (59.0)	232 (39.7)	<0.001
Diabetes, *n* (%)	338 (14.5)	355 (38.5)	336 (36.3)	235 (40.2)	<0.001
Stroke, *n* (%)	277 (11.9)	325 (35.2)	324 (35.0)	267 (45.6)	<0.001
Previous intracranial haemorrhage, *n* (%)	25 (1.1)	22 (2.4)	21 (2.3)	31 (5.3)	<0.001
Previous extracranial bleeding, *n* (%)	49 (2.1)	39 (4.2)	31 (3.3)	84 (14.4)	<0.001
Heart failure, *n* (%)	352 (15.1)	0 (0.0)	908 (98.1)	399 (68.2)	<0.001
CAD, *n* (%)	300 (12.9)	676 (73.3)	852 (92.0)	375 (64.1)	<0.001
CKD, *n* (%)	84 (3.6)	58 (6.3)	164 (17.7)	286 (48.9)	<0.001
COPD, *n* (%)	101 (4.3)	44 (4.8)	171 (18.5)	137 (23.4)	<0.001
Dementia, *n* (%)	14 (0.6)	11 (1.2)	38 (4.1)	104 (17.8)	<0.001
Liver disease, *n* (%)	41 (1.8)	31 (3.4)	34 (3.7)	67 (11.5)	<0.001
OSA, *n* (%)	50 (2.1)	31 (3.4)	51 (5.5)	24 (4.1)	<0.001
Malignancy, *n* (%)	173 (7.4)	51 (5.5)	19 (2.1)	191 (32.6)	<0.001
Anaemia, *n* (%)	79 (3.4)	31 (3.4)	23 (2.5)	424 (72.5)	<0.001
PAD, *n* (%)	56 (2.4)	182 (19.7)	116 (12.5)	154 (26.3)	<0.001
Hyperthyroidism, *n* (%)	39 (1.7)	16 (1.7)	8 (0.9)	10 (1.7)	0.331
Hypothyroidism, *n* (%)	56 (2.4)	45 (4.9)	29 (3.1)	52 (8.9)	<0.001
Smoking, *n* (%)	196 (8.5)	72 (7.9)	55 (6.0)	23 (4.0)	0.001
CHA_2_DS_2_VASc score, median [IQR]	3 [2–4]	4 [3–5]	5 [4–6]	5 [4–6]	<0.001
CHA_2_DS_2_VA score, median [IQR]	2 [2–3]	3 [2–5]	4 [3–5]	4 [3–6]	<0.001
HAS-BLED score, median [IQR]	2 [1–2]	2 [2–3]	3 [2–3]	3 [2–4]	<0.001
Type of AF, *n* (%)					<0.001
First diagnosed	360 (17.6)	135 (16.2)	147 (19.6)	97 (22.2)	
Paroxysmal	892 (43.6)	438 (52.5)	233 (31.1)	134 (30.7)	
Persistent	379 (18.5)	132 (15.8)	137 (18.3)	72 (16.5)	
Long-standing persistent	48 (2.3)	20 (2.4)	38 (5.1)	31 (7.1)	
Permanent	365 (17.9)	109 (13.1)	195 (26.0)	103 (23.6)	

**Legend**: AF, atrial fibrillation; BMI, body mass index; CAD, coronary artery disease; CKD, chronic kidney disease; COPD, chronic obstructive pulmonary disease; IQR, interquartile range; N, number; OSA, obstructive sleep apnoea; PAD, peripheral artery disease.

Almost half of the patients (*n* = 2332, 48.9%; 40.5% females) belonged to class 1, defined as the ‘low complexity’ class. These patients were the youngest among the classes (median age 74, IQR 64–80 years), had the lowest number of comorbidities (median number 2, IQR 1–2) and the lowest CHA_2_DS_2_VASc and HAS-BLED scores. Hypertension was the most common condition, present in 52.8% of these individuals, followed by lipid disorders, HF, and diabetes, diagnosed in less than one-fifth of this cohort. Paroxysmal AF was the most frequent type of AF (43.6%).

Class 2 comprised 922 (19.3%) patients (median age 76, IQR 68–82 years; 37.5% females), characterised by an ‘atherosclerotic’ phenotype. In this phenotype, there was a high prevalence of CV risk factors, such as lipid disorders (96.0%), hypertension (79.4%), and diabetes (38.5%). Similarly, the prevalence of CAD was high (73.3%), but these patients did not suffer from HF.

Class 3 (median age 81, IQR 75–85 years; 40% females) could be described as the ‘heart failure’ class. This group accounted for 19.4% (*n* = 926) of the whole cohort, and was mainly characterized by HF, diagnosed in almost the totality of these patients (98.1%), and CAD (92.0%). Conversely, the prevalence of non-CV conditions was relatively low. Of note, COPD was present in 18.5% of this cohort, showing the second highest prevalence, after class 4.

Class 4, the ‘high complexity’ phenotype, was composed of 585 patients, accounting for 12.3% of the whole cohort. This group was characterized by the most advanced age (median age 84, IQR 78–88 years), and a high prevalence of males (65.3%). Moreover, they suffered from the highest number of concomitant CV and non-CV comorbidities (median number 6, IQR 5–7); in particular, among the first ones, hypertension, HF, stroke, and CAD were the most common, while concerning non-CV conditions, the highest prevalence was observed for anaemia (72.5%), CKD (48.9%), and neoplasia (32.6%). COPD (23.4%), dementia (17.8%), and previous intracranial (5.3%) and extracranial (14.4%) bleedings were less frequent, but still showed the highest prevalence in this class, compared to the others.

Overall, there was a progressive increase in the number of comorbidities from class 1 to class 4. Thus, given the lowest burden of comorbidities in class 1 (‘low complexity’), this was used as the reference group for the following analyses.

### Treatments according to latent classes

**Figure 3:**
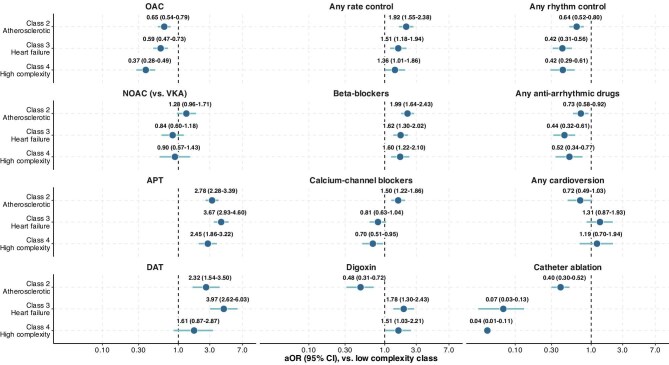
Logistic regression for treatments according to latent classes. **Legend**: aOR, adjusted odds ratio; APT, antiplatelet therapy; CI, confidence interval; DAT, dual antithrombotic therapy; NOAC, non-vitamin K antagonist oral anticoagulant; OAC, oral anticoagulant; and VKA, vitamin K oral anticoagulant. Adjusted for CHA_2_DS_2_VASc score and type of AF.

*Table 2* reports the treatments according to latent classes, while *Figure 3* shows the results of the multivariable logistic regression analyses. The use of OAC was the highest in ‘low complexity’, and progressively decreased in the ‘atherosclerotic’, ‘heart failure’, and ‘high complexity’ classes. These results were confirmed on multivariable regression analysis: compared to ‘low complexity’, all other classes showed lower odds of being prescribed with OAC (aOR, 95% CI: 0.65, 0.54–0.79 for class 2; 0.61, 0.49–0.75 for class 3; and 0.39, 0.29–0.51 for class 4), while no differences were observed concerning the type of OAC (NOAC vs. VKA) among the classes (*Figure 3*). Trends concerning the prescription of OAC within the enrolment period in the overall cohort and stratified according to latent classes are shown in Supplementary material online, *Figure S1*.

**Table 2:** Treatments according to latent classes

	Class 1	Class 2	Class 3	Class 4	
	Low complexity	Atherosclerotic	Heart failure	High complexity	*P* value
OAC, *n* (%)	1186 (50.9)	406 (44.0)	367 (39.6)	171 (29.2)	<0.001
NOAC, *n* (%)	635 (27.3)	231 (25.1)	152 (16.4)	72 (12.3)	<0.001
VKA, *n* (%)	551 (23.7)	175 (19.0)	215 (23.2)	99 (16.9)	<0.001
APT, *n* (%)	678 (29.2)	495 (53.7)	559 (60.5)	279 (47.8)	<0.001
DAT, *n* (%)	74 (3.2)	74 (8.0)	100 (10.8)	23 (3.9)	<0.001
Any rate control, *n* (%)	1359 (58.5)	712 (77.2)	717 (77.9)	452 (77.3)	<0.001
Beta blockers, *n* (%)	994 (42.8)	576 (62.5)	559 (60.7)	355 (60.7)	<0.001
Digoxin, *n* (%)	210 (9.0)	43 (4.7)	192 (20.9)	95 (16.2)	<0.001
Ca-channel blockers, *n* (%)	529 (22.8)	331 (35.9)	290 (31.4)	187 (32.0)	<0.001
Any rhythm control, *n* (%)	859 (37.0)	294 (31.9)	167 (18.1)	98 (16.8)	<0.001
Amiodarone, *n* (%)	435 (18.7)	148 (16.1)	81 (8.8)	43 (7.4)	<0.001
Propafenone, *n* (%)	172 (7.4)	46 (5.0)	13 (1.4)	12 (2.1)	<0.001
Any cardioversion, *n* (%)	215 (9.2)	91 (9.9)	103 (11.1)	65 (11.1)	0.310
Catheter ablation, *n* (%)	529 (22.8)	98 (10.6)	15 (1.6)	7 (1.2)	<0.001
ACEi/ARB, *n* (%)	807 (34.8)	411 (44.6)	429 (46.5)	223 (38.1)	<0.001
Diuretics, *n* (%)	401 (17.3)	161 (17.5)	440 (47.7)	267 (45.6)	<0.001
Oral glucose lowering drugs, *n* (%)	201 (8.7)	236 (25.8)	191 (20.7)	111 (19.0)	<0.001
Insulin, *n* (%)	81 (3.5)	83 (9.0)	105 (11.4)	80 (13.7)	<0.001
Statins, *n* (%)	949 (40.8)	817 (88.6)	693 (75.2)	336 (57.4)	<0.001
PPI, *n* (%)	369 (15.9)	145 (15.7)	199 (21.6)	172 (29.4)	<0.001

**Legend**: ACEi, angiotensin-converting enzyme inhibitor; APT, antiplatelet therapy; ARB, angiotensin receptor blocker; DAT, dual antiplatelet therapy; NOAC, non-vitamin K antagonist oral anticoagulant; OAC, oral anticoagulant; PPI proton-pump inhibitor; and VKA, vitamin K antagonist.

The use of APT, conversely, increased from the ‘low complexity’ class, towards the ‘high complexity’ class, and was the highest in the ‘atherosclerotic’ (53.7%) and ‘heart failure’ (60.5%) classes. Consistently, on logistic regression, we found that ‘high complexity’, ‘atherosclerotic’, and ‘heart failure’ classes had higher odds of receiving APT. DAT was more common in the ‘heart failure’ and ‘atherosclerotic’ classes (10.8% and 8.0%, respectively), as confirmed also on multivariable analysis shown in *Figure 3* (aOR, 95% CI: 4.03, 2.66–6.12; and 2.31, 1.53–3.47, respectively).

Rate control strategies were used in more than half of the patients included in ‘low complexity’ and approximately two thirds of patients in the other classes. Similarly, logistic regression analysis confirmed higher odds of receiving rate control strategies, despite some differences concerning the various classes of drugs detailed in *Table 2* and *Figure 3*. Regarding rhythm control approaches, their use was more frequent in the ‘low complexity’ class (37.0%), and progressively decreased in ‘atherosclerotic’ (31.9%), ‘heart failure’ (18.1%), and ‘high complexity’ (16.8%) classes. Consistently, on logistic regression, all these latent classes were less likely to receive rhythm control strategies, compared to the ‘low complexity’ one, as shown in *Figure 3*.

The use of antiarrhythmic drugs and catheter ablation mirrored the distribution of the rhythm control approaches as a whole, showing a wider use in the ‘low complexity’ class, while no differences were observed concerning electrical or pharmacological cardioversions among the classes (*Table 2* and *Figure 3*).

With respect to other treatments, diuretics were used in almost half of individuals in ‘high complexity’ and ‘heart failure’ classes, while the use of ACEi or ARB was the highest in the ‘heart failure’ and ‘atherosclerotic’ classes (*Table 2*). The prescription of statins progressively increased from the ‘low complexity’ class to the ‘atherosclerotic’ class, and finally, we also found higher use of non-CV drugs [i.e. proton pump inhibitors (PPI)] in more complex phenotypes (*Table 2*).

### Follow-up and risk of adverse events according to latent classes

During the 1-year follow-up, 435 (9.1%) events of the primary outcome were reported. The event count for the primary outcome was the lowest in the ‘atherosclerotic’ class (*n* = 37, 4.0%), progressively increased in the ‘low complexity’ class (*n* = 122, 5.2%), in the ‘heart failure’ class (*n* = 112, 12.1%), and was the highest in the ‘high complexity’ class (*n* = 164, 28.0%). Event counts for the secondary exploratory outcomes are detailed in *Table 3*.

**Table 3:** Events count, univariable and multivariable regressions on the risk of major outcomes according to latent classes

	Class 1	Class 2	Class 3	Class 4
	Low complexity	Atherosclerotic	Heart failure	High complexity
Primary composite outcome (all-cause death, CV death, TE events, ACS)				
*N* (%)	122 (5.2)	37 (4.0)	112 (12.1)	164 (28.0)
OR (95% CI)	Ref.	0.76 (0.51–1.09)	2.50 (1.91–3.26)	7.06 (5.47–9.13)
aOR* (95% CI)	Ref.	0.68 (0.41–1.09)	1.50 (1.04–2.17)	3.20 (2.21–4.66)
All-cause death				
*N* (%)	88 (3.8)	22 (2.4)	87 (9.4)	144 (24.6)
OR (95% CI)	Ref.	0.62 (0.38–0.98)	2.64 (1.94–3.60)	8.33 (6.28–11.10)
aOR* (95% CI)	Ref.	0.49 (0.25–0.90)	1.37 (0.89–2.10)	3.59 (2.38–5.43)
CV death				
*N* (%)	31 (1.3)	7 (0.8)	38 (4.1)	24 (4.1)
OR (95% CI)	Ref.	0.57 (0.23–1.22)	3.18 (1.97–5.17)	3.18 (1.83–5.44)
aOR* (95% CI)	Ref.	0.64 (0.18–1.80)	2.64 (1.29–5.52)	1.83 (0.72–4.45)
TE events				
*N* (%)	27 (1.2)	11 (1.2)	27 (2.9)	22 (3.8)
OR (95% CI)	Ref.	1.03 (0.49–2.03)	2.57 (1.50–4.43)	3.39 (1.90–5.99)
aOR* (95% CI)	Ref.	1.76 (0.77–3.94)	2.01 (0.96–4.28)	2.19 (0.94–5.02)
ACS				
*N* (%)	17 (0.7)	7 (0.8)	15 (1.6)	11 (1.9)
OR (95% CI)	Ref.	1.04 (0.40–2.42)	2.25 (1.11–4.54)	2.65 (1.20–5.63)
aOR* (95% CI)	Ref.	0.66 (0.15–2.16)	1.54 (0.58–3.94)	1.23 (0.32–3.90)
Major bleeding				
*N* (%)	18 (0.8)	6 (0.7)	14 (1.5)	40 (7.0)
OR (95% CI)	Ref.	0.84 (0.30–2.01)	1.98 (0.96–3.99)	9.60 (5.55–17.28)
aOR* (95% CI)	Ref.	0.83 (0.26–2.23)	1.06 (0.39–2.66)	4.28 (1.96–9.58)

**Legend**: ACS, acute coronary syndrome; aOR, adjusted odds ratio; CI, confidence interval; CV, cardiovascular; N, number; OR, odds ratio; and TE, thromboembolic.

*Adjusted for: age, sex, use of OAC, type of AF.

Compared to the ‘low complexity’ class, both the ‘high complexity’ and the ‘heart failure’ classes showed a higher risk of adverse events of the composite outcome, and these results were confirmed on the adjusted model (aOR, 95% CI: 3.20, 2.21–4.66; and aOR, 95% CI 1.50, 1.04–2.17, respectively). However, no statistically significant difference was observed for the ‘atherosclerotic’ class (*Table 3*). Regarding all-cause death, ‘high complexity’ and ‘heart failure’ classes were associated with a higher risk compared to the ‘low complexity’ class; conversely, the ‘atherosclerotic class’ was associated with a lower risk. These results were partially confirmed on multivariable regression, the ‘high complexity’ class was associated with a higher risk (aOR, 95% CI: 3.59, 2.38–5.43), the ‘atherosclerotic’ class with a lower risk (aOR, 95% CI: 0.49, 0.25–0.90), whereas only a trend towards a higher risk was observed for the ‘heart failure’ class (*Table 3*).

Concerning CV death, despite a higher risk on unadjusted analysis for the ‘high complexity’ and the ‘heart failure’ classes, after adjusting for possible confounders, only the ‘heart failure’ class showed statistically significant association with this outcome (aOR, 95% CI: 2.64, 1.29–5.52). Conversely, the ‘high complexity’ class was the only subgroup of patients associated with a significantly higher risk of major bleeding (aOR, 95% CI: 4.28, 1.96–9.58). The risk of TE events was higher for the ‘high complexity’ and the ‘heart failure’ classes, whereas only a nonstatistical trend towards a higher risk was observed after adjusting for covariates. Similarly, concerning the risk of ACS, despite a higher risk for ‘heart failure’ and ‘high complexity’ on univariable regression, no statistically significant differences were observed on multivariable analysis (*Table 3*).

### Adherence to the ABC pathway

From the whole cohort of patients included in the main analysis, 2654 (55.7%) patients (median age 75, IQR 66–81 years; 43.3% females) with available information to assess the adherence to the ABC pathway were included in this analysis. Compared to patients not included due to missing data, included patients were younger (median age 75, IQR 66–81 vs. 80, IQR 72–85), and more frequently females (43.3% vs. 33.8%).

Among these patients, 1094 (41.2%) were ABC pathway adherent. In detail, the greatest adherence to the ABC pathway was observed in the ‘atherosclerotic’ class (44.1%), followed by the ‘low complexity’ class (42.6%), the ‘heart failure’ class (38.8%), and was the lowest in the ‘high complexity’ class (30.8%) (*Table 4*). Compared to nonadherent, ABC adherent patients were associated with a lower risk of adverse events of the primary composite outcome (aOR, 95% CI: 0.37, 0.20–0.65), and all-cause death (aOR, 95%vCI: 0.32, 0.13–0.68), as described in Supplementary material online, *Table S1*. No statistically significant differences were observed concerning the other secondary exploratory outcomes (Supplementary material online, *Table S1*). When grouping according to the number of adherent criteria, compared to patients adherent to 0–1 ABC criteria, no differences were found in patients adherent to 2 criteria regarding all the outcomes, despite a trend towards a lower risk concerning all-cause death. Conversely, patients adherent to 3 criteria were associated with a lower risk of the primary outcome, all-cause death, and CV death on univariable analysis, and these results were confirmed for the primary outcome and all-cause death, after adjusting for covariates (Supplementary material online, *Table S1*).

**Table 4:** Adherence to ABC according to latent classes

	Class 1	Class 2	Class 3	Class 4	
	Low complexity	Atherosclerotic	Heart failure	High complexity	*P* value
% of the main analysis class cohort	57.5	65.7	50.3	41.0	
*N* (%) of this ABC analysis cohort	1342 (50.6)	606 (22.8)	466 (17.6)	240 (9.0)	
ABC compliant, *n* (%)	572 (42.6)	267 (44.1)	181 (38.8)	74 (30.8)	0.002
ABC groups					<0.001
0–1 ABC criteria adherent, *n* (%)	164 (12.2)	11 (1.8)	55 (11.8)	24 (10.0)	
2 ABC criteria adherent, *n* (%)	606 (45.2)	328 (54.1)	230 (49.4)	142 (59.2)	
3 ABC criteria adherent, *n* (%)	572 (42.6)	267 (44.1)	181 (38.8)	74 (30.8)	

**Legend**: ABC, atrial fibrillation better care; and *N*, number.

**Figure 4:**
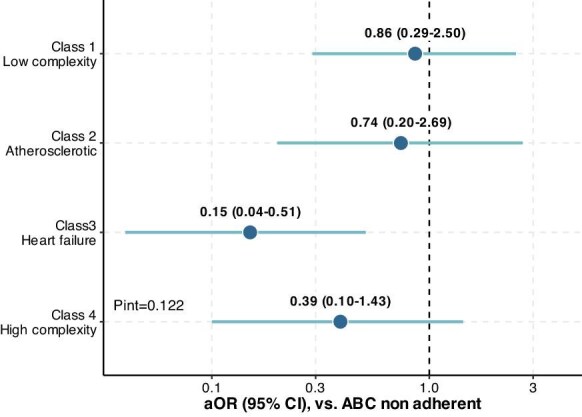
Interaction analysis between adherence to ABC and different subgroups for the primary outcome. **Legend**: ABC, atrial fibrillation better care; aOR, adjusted odds ratio; and CI, confidence interval. Adjusted for CHA_2_DS_2_-VASc score, and type of AF.

On the interaction analysis, no statistically significant interaction was found between the adherence to the ABC pathway and latent classes (Pint = 0.122), despite a more diluted effect in the ‘low complexity’, ‘atherosclerotic’, and ‘high complexity’ classes (*Figure 4*).

## Discussion

In this analysis from a large cohort of elderly Chinese AF patients, we identified four different clinical phenotypes: ‘low complexity’, ‘atherosclerotic’, ‘heart failure’, and ‘high complexity’. Second, these phenotypes were associated with differences in clinical management, including the use of OAC, anti-platelets therapies, and the choice of rate or rhythm control strategies, as well as different risks of adverse events. Third, adherence to the ABC pathway varied across clinical phenotypes, with the lowest adherence observed in more complex phenotypes; however, the beneficial impact of full adherence to the ABC pathway was similar across all the clinical phenotypes identified.

In this study, we evaluated a cohort of AF patients that was mainly composed of patients aged ≥75 years. While we named class 1 as ‘low complexity’ due to the relatively lower prevalence of comorbidities compared to other classes, none of the classes can be accurately defined as low risk, as all patients had a CHA_2_DS_2_-VASc score of ≥2. The second phenotype was named ‘atherosclerotic’, due to the high proportion of CV risk factors, and CAD, but a low proportion of other conditions, and it reflects the known role of pro atherosclerotic risk factors in the epidemiology of AF and ischaemic heart disease.^34^

The identification of a distinct ‘heart failure’ class in our cohort, not reported in cluster analyses from Western populations but described in previous Japanese studies,^35,36^ highlights regional differences in the epidemiology and pathophysiology of HF in Asia. In Asian populations, HF tends to present earlier, often by about a decade, compared to Western populations, and is associated with worse prognosis.^37^ Additionally, the high prevalence of CAD in this phenotype aligns with evidence suggesting this is a more dominant cause of HF in Asia.^38^ Furthermore, the presence of COPD in ∼20% of patients in this phenotype underscores the role of coexisting respiratory diseases, forming a triad (AF-HF-COPD) that significantly affects prognosis.^39^ These findings suggest that the ‘heart failure’ phenotype in Asian populations may represent a unique interaction of metabolic, CV, and pulmonary conditions that differs from those observed in Western cohorts.

Finally, a distinct class with a high prevalence of both CV and non-CV comorbidities has been distinguished. These patients were the most clinically complex, due to the intertwined presence of conditions like malignancy, anaemia, CKD, dementia, and previous bleeding, together with CV diseases, that exponentially complicate their management. Our results corroborate findings from prior cluster analyses performed in both Asian and Western cohorts regarding the presence, also in a cohort of Asian elderly patients, of a particularly frail and complex phenotype.^23,40,41^

Management approaches varied significantly across the clinical phenotypes. Compared to the ‘low complexity’ phenotype, other phenotypes showed lower odds of receiving OAC. Previous studies showed that the prescription of OAC decreases as the number of comorbidities increases,^42^ despite the proven clinical benefit of OAC also in very elderly and multimorbid patients.^43,44^ Physicians might have been more confident in prescribing OACs in less complex patients, fearing, in more complex phenotypes, bleeding complications due to CKD, malignancy, anaemia, and previous bleeding, or poor adherence, or preferring APT, despite its known contraindication for stroke prevention in AF patients.^16^ In contrast, DAT use was higher in the ‘heart failure’ and ‘atherosclerotic’ classes, likely due to the prevalence of CAD and recent revascularization procedures. Regional variations in antithrombotic treatment were also evident, with Asian patients generally less likely to receive OAC therapy and more likely treated with APT.^45,46^

Moreover, rate control was predominantly used, particularly in more complex patients, while the chances of receiving rhythm control approaches were notably low in all phenotypes except the ‘low complexity’ group. It appears that the degree of complexity markedly influenced the access to AF ablation, whose technology and safety had an important improvement in the last decade, in parallel with an increased implementation in practice.^47,48^ Of note, despite a quite similar age, the proportion of patients treated with AF ablation in the ‘atherosclerotic’ class, where AF was paroxysmal in 52.5% of cases, was half that of the ‘low complexity’ class. In the HF phenotype, the use of AF ablation for rhythm control was extremely low, and this should be interpreted according to the study period, before the currently available evidence in support of AF ablation to improve survival and reduce HF hospitalizations.^49^ In light of the recent results underscoring the beneficial effects of early rhythm control concerning not only symptom, but also adverse events reduction in HF patients and in patients with a high burden of comorbidities, a shift towards this approach is advisable in contemporary practice.^50–52^

Adverse event risks differed across phenotypes. The ‘low complexity’ and ‘atherosclerotic’ classes had comparable outcomes, likely due to effective secondary prevention strategies, stricter medicalization and the highest adherence to the ABC pathway in the ‘atherosclerotic’ phenotype.

Conversely, ‘heart failure’ and ‘high complexity’ classes showed the highest risk of the primary composite outcome, in line with previous reports, which reported a progressive increase in the risk of death and major adverse cardiovascular events (MACE) in patients with multimorbidity and HF.^2,5,53^ Nevertheless, some heterogeneities regarding secondary exploratory outcomes were noted. In particular, in the ‘heart failure’ phenotype, the increased risk of the composite outcome was mainly driven by a higher risk of CV death, while in the ‘high complexity’ phenotypes the main driver was all-cause death. These patients presented with numerous comorbidities, including CV and also non-CV conditions (i.e. CKD, malignancy, and anaemia), which collectively exacerbated their vulnerability and contributed to all-cause death by weakening overall health and increasing susceptibility to competing risks, such as major bleeding.

In recent years, there has been growing awareness of the importance of using integrated care approaches to minimize the risk of adverse events in AF patients.^7^ The first pioneering approach, the evidence-based ABC pathway, was later adopted and acronym renamed (but untested in clinical trials) by the latest American guidelines as S.O.S. (stroke prevention, optimization of all modifiable risk factors, and symptom management) and by the European guidelines as AF-CARE.^17,54^ The effectiveness of holistic management of AF patients (i.e. through the ABC pathway) has already been proven in this cohort and others,^10,11,13,55^ and, in the present analysis, we found consistent results. However, adherence to an integrated approach remains suboptimal, with nearly 60% of this cohort being nonadherent. Furthermore, adherence decreases as patient complexity increases, suggesting that holistic management becomes more challenging in more complex cases. Of note, the relationship between patient complexity, suboptimal management, and outcomes is also self-perpetuating. Poor adherence to the ABC pathway in these patients often leads to suboptimal management, which exacerbates disease progression. This worsening clinical status further complicates treatment decisions, creating a vitious cycle of adverse outcomes. For example, the high prevalence of anaemia, malignancy, and CKD in the ‘high complexity’ phenotype increases the likelihood of major bleeding events, discouraging physicians from prescribing OACs, thus leading to an underuse of effective stroke prevention strategies.

Our results suggest that the beneficial impact of the ABC pathway on the risk of adverse events is consistent across all different clinical phenotypes. Thus, a holistic management is of utmost importance. Moreover, the role of socioeconomic factors, the importance of frailty and the access to healthcare resources should be emphasised, since these aspects can contribute to improve or worsen outcomes.

Therefore, promoting evidence-based practices is essential to improving the management of AF, especially in complex patients. Physicians may need greater confidence in prescribing OACs and early rhythm control strategies, when appropriate, to high-risk populations, given the robust evidence of their safety and benefits. To overcome barriers faced particularly in complex cases, new tools (i.e. digital technology with smartphone applications, artificial intelligence tools, and implementation of telemedicine) could simplify adherence to integrated care pathways, especially for patients with high-complexity phenotypes, while maintaining the flexibility to tailor approaches based on the specific needs of diverse patients. Similarly, community engagement, educational programmes and awareness about AF both among healthcare providers and the general population might be helpful to improve management and treatment adherence. Moreover, addressing geographical, socioeconomic, and healthcare disparities in access to treatments, and thus to the ABC pathway, is equally crucial to ensuring equitable care.

## Limitations

Some limitations should be acknowledged while interpreting these results. The main limitation is due to the observational design of the study, thus results provide associations, but do not imply causality. Second, not all patients had complete available data to be included in the LCA analysis, thus some selection bias might have occurred.

Moreover, we selected a set of available comorbidities defined according to the electronic case records of the ChiOTEAF Registry relevant to the natural history of AF, but the potential contribution of other unrecorded diseases was not analysed and is a limitation. The enrolment period was before the guideline-recommended implementation of the holistic and integrated management of AF, and before the widespread use of some classes of medications (i.e. sodium-glucose cotransporter 2 inhibitors, angiotensin receptor/neprilysin inhibitors). Therefore, it cannot be excluded that differences in treatment could have partially changed the results. Similarly, this registry was run in the era of NOACs just into China. Thus, the use of NOACs may reflect doctors’ caution on NOACs, as they would like to use these ‘new’ drugs in relatively ‘safe’ settings. Only a subgroup of patients had available data to assess the adherence to the ABC pathway, thus our results cannot be fully generalized, although the effectiveness of the ABC pathway in many cohorts has already been well proven. Finally, despite adjusting for covariates, the effect of residual confounding factors cannot be excluded (i.e. geographic origin, socioeconomic status, healthcare access, and frailty status might have influenced management and prognosis, especially in high complex phenotypes).

## Conclusion

In elderly Asian patients with AF, different clinical phenotypes, each characterized by unique patterns of comorbidities, can be clearly distinguished. These heterogeneous phenotypes significantly influenced both management strategies and patient prognosis. Adherence to the ABC pathway was shown to improve outcomes for Asian AF patients, and this beneficial impact was similar across the various clinical phenotypes.

## Supplementary Material

qcaf014_Supplemental_File

## Data availability

The data underlying this article will be shared on reasonable request to the corresponding author.

## Notes

ChiOTEAF Registry Investigators are listed in Supplementary material.
